# Acute kidney injury: short-term and long-term effects

**DOI:** 10.1186/s13054-016-1353-y

**Published:** 2016-07-04

**Authors:** James F. Doyle, Lui G. Forni

**Affiliations:** Department of Intensive Care Medicine and Surrey Peri-Operative Anaesthesia and Critical Care Collaborative Research Group, Royal Surrey County Hospital NHS Foundation Trust, Egerton Road, Guildford, GU2 7XX Surrey UK; Faculty of Health and Medical Sciences, University of Surrey, Guildford, Surrey UK

## Abstract

Acute kidney injury (AKI) is the most common cause of organ dysfunction in critically ill adults, with a single episode of AKI, regardless of stage, carrying a significant morbidity and mortality risk. Since the consensus on AKI nomenclature has been reached, data reflecting outcomes have become more apparent allowing investigation of both short- and long-term outcomes.

Classically the short-term effects of AKI can be thought of as those reflecting an acute deterioration in renal function per se. However, the effects of AKI, especially with regard to distant organ function (“organ cross-talk”), are being elucidated as is the increased susceptibility to other conditions. With regards to the long-term effects, the consideration that outcome is a simple binary endpoint of dialysis or not, or survival or not, is overly simplistic, with the reality being much more complex.

Also discussed are currently available treatment strategies to mitigate these adverse effects, as they have the potential to improve patient outcome and provide considerable economic health savings. Moving forward, an agreement for defining renal recovery is warranted if we are to assess and extrapolate the efficacy of novel therapies. Future research should focus on targeted therapies assessed by measure of long-term outcomes.

## Background

Acute kidney injury (AKI) is the most common cause of organ dysfunction in critically ill adults with an incidence of around 34 %, and carries an observed in-hospital mortality as high as 62 % [1]. Indeed, even a single episode of AKI carries a significant morbidity and mortality risk, with an episode of stage 1 AKI complicating a critical illness being independently associated with an increase in 10-year mortality [2]. Moreover, the long-term effects of AKI also contribute significantly to health costs and, in the UK, has been estimated to account for about 1 % of the total health and social care budget.

In this narrative review we will consider how, since a consensus has been reached, the effects of kidney injury on both short- and long-term outcomes have been highlighted.

## Defining renal injury

Table 1 describes the terminology used to describe and define AKI throughout its course from that described by the Kidney Disease Improving Global Outcomes (KDIGO) and others [3, 4].

**Table 1:** Defining renal injury

Renal Injury	Description
Acute kidney injury (AKI)	Defined according to changes in serum creatinine and urine output, and described as stages 1, 2 and 3
Recurrent kidney injury (RKI)	An episode of repeated renal injury following recovery of the index case
Acute kidney disease (AKD)	Describes the transition between the acute and chronic states (day 7 post-AKI to day 90)
Chronic kidney disease (CKD)	Abnormalities of kidney structure or function for >3 months, with implications for health

The definition of chronic kidney disease (CKD) as published by the KDIGO guidelines is outlined in Table 1, with the latest review including ‘with implications for health’ in an attempt to contextualize the notion that a variety of kidney functional anomalies may exist that will not have bearing on the patient’s health long term. CKD has then been sub-classified based on cause, estimated glomerular filtration rate (eGFR), or albuminuria category into stages 1–5 [3].

Perhaps the most challenging area to define is that of renal recovery; however, this is of vital importance, as a consensus agreement would likely yield a framework for efficacy of assessment of novel emerging therapies. However, it is not without difficulty. Within the critical care arena some would consider renal recovery as the return of eGFR to pre-morbid baseline levels (whatever that means in the context of critical illness) whilst others may consider the absence of the need for renal replacement therapy (RRT) as sufficient recovery. Studies reporting renal recovery are often ambiguous at best. There has been some attempt at the classification of renal recovery [5] (Table 2).

**Table 2:** Potential definition of renal recovery

Renal recovery	Assessment
Complete	Return of pre-AKI renal function, assessed as eGFR ±10 % of baseline
Partial	Persistent impaired eGFR, but no requirement for RRT
Absent	Persistent requirement for RRT

Data adapted from Schiffl and Fischer [5]. *AKI* acute kidney injury, *eGFR* estimated glomerular filtration rate, *RRT* renal replacement therapy

However, using a crude marker of renal function such as serum creatinine outside steady-state conditions has significant limitations. Serum creatinine is used as a surrogate for the glomerular filtration rate (GFR) during AKI in critically ill unstable patients. The GFR is rarely measured in clinical practice but equations, such as the modification of diet in renal disease (MDRD) and the chronic kidney disease epidemiology collaboration (CKD-EPI) equations, estimate the GFR (eGFR) from the serum creatinine where differences in age, sex, and race are considered [6–8]. However, as noted, creatinine is a biomarker for the GFR only under clinically stable conditions [9]. In the critically ill there is a significant overestimation of the eGFR due to, for example, reduced creatinine levels due to reduced muscle bulk and creatinine generation. Several studies have compared formal measurement of the GFR and eGFR with considerable disparity observed [10]. Most importantly, of course, is that there is a delay between renal injury and any observed rise in creatinine, which may be masked for up to 48 h.

## Risk prediction

**Fig. 1:**
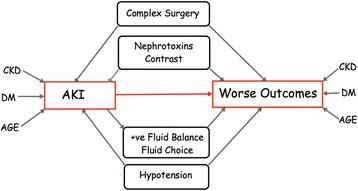
Influences on outcome for AKI. Influences of various co-morbidities on the development of AKI, and unfavourable long-term outcomes. *AKI* acute kidney injury, *CKD* chronic kidney disease, *DM* diabetes mellitus

In terms of outcomes from AKI, various factors may influence both short- and long-term sequelae (Fig. 1). For example, the presence of pre-existing CKD has a significant impact on long-term outcomes. One retrospective study demonstrates that CKD not only predisposes an individual to a higher risk of AKI but also longer duration of RRT, and more in-hospital resuscitation was observed in this group. Indeed, eGFR was an independent predictor of both 30-day mortality (hazard ratio (HR) 0.994, 95 % confidence interval (CI) 0.990–0.998) and 1-year mortality (HR 0.996, 95 % CI 0.993–1.000) [11]. Unsurprisingly, other risk factors for the development of AKI, such as age and diabetes, are also associated with worse outcomes. In patients with AKI aged over 65 years when matched to a younger cohort worse outcomes were found at 3 months, although the longer term outcome was independent of age [12]. Clearly, the severity of any concomitant illness will also play a considerable role in outcome and ultimately decide prognosis despite the improvement in supportive organ support for critically unwell patients [13]. Similarly, the increasing complexity of procedures offered to patients with significant comorbidities is increasing with a rise in AKI. Patients undergoing coronary artery bypass grafting have a risk of AKI in one series of 2.5 % compared to 4.6 % of patients undergoing valvular surgery [14]. Exposure to nephrotoxins, including contrast, also plays a role in determining outcome; contrast administration is associated with increased need for RRT, worse renal function at discharge, and longer length of stay, as well as worse short-term mortality [15]. Furthermore, the choice of resuscitation fluid employed can also have significant consequences as is the case for some hydroxyethyl starch (HES) derivatives, where increased need for RRT and increased mortality in patients that were resuscitated with HES has been observed [16, 17]. Risk assessment may be enhanced by the use of scoring systems such as the Sequential Organ Failure Assessment (SOFA) and the Acute Physiology and Chronic Health Evaluation (APACHE), of which there are several variations. For example, the occurrence of a concurrent AKI and SOFA score >11 confers a significant negative short-term outcome [1]. There are considerable data on the development of AKI, particularly in the surgical patient; however, no system is uniquely placed in terms of predictive ability. Consequently, much effort has been focused on the development of ‘biomarkers’ for AKI in order to enhance early detection [18]. Many studies have utilized biomarkers alone or in combination with scoring systems and, recently, combination biomarkers of cell cycle arrest: tissue inhibitor of metalloproteinases (TIMP)-2/insulin-like growth factor binding protein (IGFBP)-7, together with urine output and serum creatinine, has been validated to be superior to predicting dialysis requirements at 9 months in patients with AKI compared to creatinine alone [19–22]. Interestingly, some biomarkers have been observed to remain elevated years after an episode of AKI, although it is unclear whether this translates into continuous pathological insult. This is an obstacle in considering novel methods employing biomarkers in terms of defining renal recovery.

## Short-term effects of AKI

**Fig. 2:**
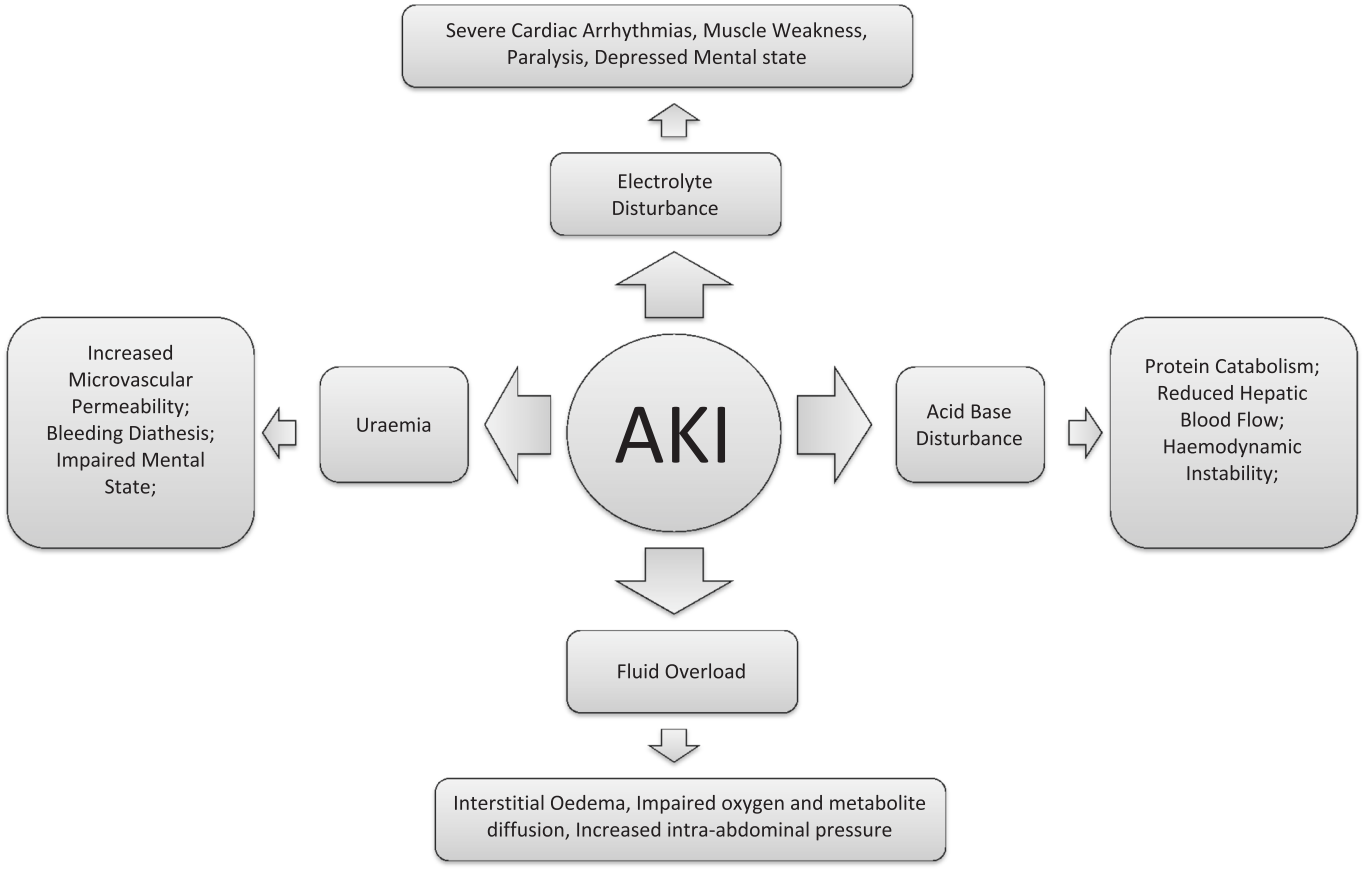
Classical short-term effects of AKI. Short-term effects have always been considered those that occur as a consequence of the acute deterioration in renal function. *AKI* acute kidney injury

Classically, the short-term effects of AKI can be thought of as those reflecting an acute deterioration in renal function per se, and include disturbances in acid-base and electrolyte homeostasis, uraemia and the consequences thereof, as well as volume overload through salt and water retention complicating AKI in many patients. This is outlined in Fig. 2. Increasingly, the effects of AKI, especially with regard to distant organ function (so called “organ cross-talk”), is being elucidated as is the increased susceptibility to other conditions, although it can be difficult to determine the exact relationship between cause and effect. An example of this is sepsis, where the presence of AKI, through effects on the innate immune system, predisposes to an episode of sepsis; this may in turn exacerbate the severity of AKI.

AKI is often associated with volume overload and this seems to be a major contributing factor to both morbidity and mortality. Indeed, a retrospective analysis of post-operative percentage fluid overload (PFO) in patients at AKI stage 3 after cardiac surgery illustrated this with a PFO >7.2 % throughout the intensive care unit (ICU) stay being significantly associated with reduced 90-day survival (*P* < 0.001) [23]. As a consequence, instigation of RRT, at least in the ICU setting, is often triggered by oliguria and volume overload despite a paucity of data supporting this approach. Further support for this approach comes from a prospective multi-centre observational study in patients with AKI where a positive fluid balance at initiation of RRT of >3 L/24 h (odds ratio (OR) 2.3, 95 % CI 1.2–4.5) and PFO >5 % (OR 2.3, 95 % CI 1.2–4.7) was associated with higher mortality [24]. Therefore, it would appear that limiting volume overload is associated with improved outcomes, and that early initiation of RRT may confer a survival benefit. To date there is no evidence from randomised controlled studies that such an approach, albeit attractive, is advantageous. Also, early instigation of treatment must be balanced against the risks associated with commencing such an invasive technique.

### AKI: a systemic disease?

As pointed out, as well as the conventional clinical features associated with AKI there is also increasing evidence that this syndrome is associated with an increased risk of developing further complications. The PICARD study, an observational multi-centre study of 618 critically ill patients showed that 40 % went on to develop sepsis after AKI had occurred. Compared to those without sepsis but with AKI, there was an increase in length of stay (LOS; 37 vs 27 days; *P* < 0.001), risk of dialysis requirement (70 vs 50 %; *P* < 0.001) and mortality (44 vs. 21 %; *P* < 0.0001) [25]. Such observations have led to a considerable body of evidence, principally from animal and laboratory work, highlighting the effect of AKI on the immune system. Various mechanisms have been proposed to explain this phenomenon including reduced pro-inflammatory cytokine clearance [26] and impaired neutrophil function [27]. It follows that such sequelae will have consequences over and above that of changes in urine output and serum creatinine, and, as such, AKI is associated with multi-system effects. This systemic approach to AKI is reflected in other short-term effects. An episode of AKI confers an increased risk of further episodes of AKI, perhaps through the mechanisms described, the subsequent development of acute kidney disease (AKD), and ultimately the risk of CKD. In some studies, recurrent kidney injury was seen in up to 31 % of patients who had higher hospital and 1-year mortality [28]. LOS and hospital re-admission are also associated with the development of AKI. Studies from the UK demonstrate that LOS has been reported as 1.5-, 1.9-, and 2.2-fold greater in those with Acute Kidney Injury Network (AKIN) 1, AKIN 2, and AKIN 3 [29]. Moreover, the same study reported an increase in hospital re-admission rate, with an increased 30-day re-admission (OR 1.93, 95 % CI 1.75–2.13) irrespective of severity of AKI [29]. The major short-term effect of AKI is, of course, mortality. An increase in hospital, ICU, 30- and 90-day mortality following AKI has been repeatedly shown, and the mortality effects of this clinical syndrome are now generally accepted. In a large UK retrospective observational database study extrapolated to an entire hospital setting of over 36,000 admissions, in-hospital mortality was recorded as non-AKI 2.0 %, AKI-1 8.1 %, AKI-2 25.6 %, and AKI-3 33.3 % [29]. Another retrospective cohort study on severity of AKI (not requiring dialysis) with a 90-day follow-up showing progression to stage 3, 4, and 5 CKD increased the risk of mortality by 0.7 %, 2.3 %, and 4.1 %, respectively [7]. This observation is consistent across different patient groups. For example, in critically unwell trauma patients, the rate of 30-day mortality was 17.5 % in an AKI cohort versus 5.8 % in the non-AKI cohort in a large observational trial in Sweden [30].

## Long-term effects of AKI

Until fairly recently, follow-up studies of survivors of critical care have focused, primarily, on short-term outcomes, such as 28-day mortality or length of hospital stay. This is of particular relevance when one considers the effects of AKI. Rather than the outcome being a simple binary endpoint of dialysis or not, or survival or not, the picture is much more complex and worrying than that. If one considers some of the economic burden of the treatment of AKI, for example, this is considerable. Indeed, observational studies demonstrate a significant deterioration of renal function in patients that have survived an episode of AKI with an apparent initial resolution, measured as eGFR. Follow-up from a retrospective observational cohort in 2012 showed progression of renal impairment by 90 days post-index AKI [7]. Furthermore, a prospective observation study of a cohort of 226 survivors of AKI patients treated with RRT found that 14 % had CKD at the end of the 5-year follow-up period [5] The long-term effects on renal function are common even where “renal recovery”, as determined by return of eGFR to pre-morbid levels, has occurred. Persistent microalbuminuria for periods as long as 4 years post-AKI have been observed [31]. The effect of an episode of AKI was elegantly demonstrated by Kolonko et al., who observed that the long-term risk for graft loss was significantly higher among the group of kidneys recovered from donors with AKI than those without (27.8 % vs 7.1 %; *P* = 0.02) [32]. Interestingly, the need for long-term dialysis is also related to AKI. The RENAL study follow-up demonstrated 5.4 % long-term requirement for maintenance dialysis after an episode of AKI requiring RRT, with Schiffl and Fischer reporting a similar incidence of 5 % [5, 31]. A pooled analysis by Goldberg and Dennen found a wide range of results, depending on patient population, but an average long-term dialysis requirement of 12.5 % in survivors over follow-up periods of 1 to 10 years [33]. In keeping with a long-term risk for chronic RRT, patients surviving AKI also have an increased rate of both adverse cardiovascular and cerebrovascular events. In a large, matched cohort of over 4000 patients, the incidence of coronary events were 19.8 and 10.3 per 1000 person years in the AKI and the non-AKI group, respectively (HR 1.67, 95 % CI 1.36–2.04), and the incidence of stroke was both higher (HR 1.25; *P* = 0.037) and of increased severity in the AKI group [34]. Following on from these observations, it would seem likely that quality of life (QOL) indices also show a relationship with AKI. A prospective, matched cohort study compared long-term outcome and QOL in patients with AKI requiring RRT compared to matched controls using the EuroQOL-SD and Short-Form (SF)-36 before ICU admission, then at 3 months, 1 year and 4 years after ICU discharge. Surprisingly, despite a higher severity of illness and 28.6 % remaining dialysis-dependent in the AKI-RRT group, the QOL scores were comparable to non-AKI RRT patients. Interestingly, both groups had worse QOL scores than the general public. As expected, the lowest QOL level was at 3 months and then improved, although still below baseline, at 1 and 4 years [35]. These results were mirrored by data from the FINNAKI study, with non-significant differences in EuroQOL-SD measurement at 6 months between AKI and non-AKI ICU patients [36]. However, despite these observations, it is known that in the USA an episode of AKI confers an OR of 2 for the requirement of transfer from hospital to a long-term care facility [37].

As discussed, an episode of AKI carries a significant short-term mortality risk depending on the severity of AKI, and this relationship also holds for longer term outcomes. In an extended follow up of the RENAL study, there were 810 survivors form the original RENAL study at day 90, of which 32 % died during the 4-year follow-up period, reflecting an overall mortality rate of 62 % in the study cohort [31]. In keeping with these results, the FINNAKI study reported 6-month mortality at 35.5 % for AKI patients and 16.5 % for non-AKI patients admitted with a critical illness [36]. A recent prospective cohort evaluation of 2010 ICU patients in a tertiary centre revealed an adjusted 10-year mortality risk of 1.26 (1.0–1.6). After propensity matching, even stage 1 AKI was associated with decreased 10-year survival (*P* = 0.036) [2].

These long-term outcomes for AKI repeatedly demonstrate high mortality rates; for example, Morgera and Hans, in 979 RRT-requiring AKI patients: 14 % 5-year survival [13]. Interestingly, careful analysis of mortality outcome has revealed a biphasic pattern [38]: an initial high rate of mortality in the immediate post-AKI setting followed by a steady, but increased, mortality rate against matched non-AKI controls.

It must be remembered that AKI is not a clinical diagnosis but a clinical syndrome characterized by a cluster of observations. The causes of AKI are numerous, and therefore it comes as no surprise that the varying aetiology of AKI may also affect long-term outcome. This is seen in a single-centre analysis of mortality after AKI at 6 months and 5 years. Only a small increase in mortality from 6 months to 5 years was seen (10 %), potentially explained by a higher than usual contribution due to intoxication and rhabdomyolysis. Regardless, the total all-cause 5-year mortality was high at 65 % and consistent with other long-term outcome AKI studies [39]. Finally of note is the fact that the development of advanced CKD, dialysis dependence, and mortality are competitive endpoints, as many patients surviving AKI do not live long enough to develop advanced CKD requiring dialysis, for example.

## Treatment options to limit the effects of AKI

The development of unique treatments during an episode of AKI to mitigate the observed short- and long-term effects have not met with success. It is likely that a combination of therapeutic strategies will result in the best outcome given the multifactorial nature of AKI. The components of these strategies are now being recognized and may include rapid detection and management as well as choice of treatment, which may well impact on both short- and long-term outcomes. Such a relationship is reflected in the mode of RRT used during AKI, which has been shown to affect long-term outcome. A retrospective cohort study in Canada of over 4000 propensity-matched patients undergoing intermittent dialysis compared to those on continuous renal replacement therapy (CRRT) showed that the risk of long-term dialysis-dependence was significantly reduced in the group of patients that initially received CRRT (HR 0.75, 95 % CI 0.65–0.87). This result was even more significant in those with pre-existing CKD or heart failure [40]. Interestingly, these results are not consistent with the recently released 4-year extended follow-up of the RENAL study in which there was no benefit of higher dose RRT in terms of mortality or long-term dialysis-dependence [31]. However, there appears to be clear benefit with the use of CRRT compared to intermittent RRT (IRRT) as an independent predictor of renal recovery [41]. Although there may be a survival benefit where CRRT is used, there is little evidence that the timing of RRT has any effects on outcome. A recent retrospective review from China demonstrated no difference in outcome with early versus late RRT [42].

Perhaps the most important consideration in terms of long-term follow-up is that treatment should remain in-line with the management of CKD.

As such, mitigation of long-term effects should include:

1. Referral to a nephrology specialist, with evidence demonstrating a reduced all-cause mortality in patients with severe AKI who undergo RRT. In addition, early referral in patients with CKD also demonstrates a survival benefit [43, 44].
2. Control of hypertension with manipulation of the renin-angiotensin-aldosterone system.
3. Heavy proteinuric states should have more aggressive control of hypertension and use of anti-proteinuric agents.
4. Consideration of the high-risk nature of developing AKI in those with CKD during critical illness.
5. General cardiovascular health, lifestyle, and dietary advice.

## Conclusion

The finding that critically unwell patients with AKI who survive the index case continue to have increased morbidity and mortality long-term remains unexplained, but is significant. Future research should focus on these patients with targeted therapies assessed by measure of long-term outcomes.

A consensus agreement for defining renal recovery is warranted if we are to assess and extrapolate the efficacy of novel therapies. Currently available treatment strategies have the potential to improve patient outcome and provide considerable economic health savings provided they are adopted early in the course of the patient’s illness with appropriate specialist follow-up to be recommended.

## Abbreviations

AKI, acute kidney injury; AKIN, Acute Kidney Injury Network; APACHE, Acute Physiology and Chronic Health Evaluation; CI, confidence interval; CKD, chronic kidney disease; CRRT, continuous renal replacement therapy; eGFR, estimated glomerular filtration rate; GFR, glomerular filtration rate; HES, hydroxyethyl starch; HR, hazard ratio; ICU, intensive care unit; KDIGO, Kidney Disease Improving Global Outcomes; LOS, length of stay; OR, odds ratio; PFO, percentage fluid overload; QOL, quality of life; RRT, renal replacement therapy; SOFA, Sequential Organ Failure Assessment.
